# Dual RNA-Seq reveals transcriptionally active microbes (TAMs) dynamics in the serum of dengue patients associated with disease severity

**DOI:** 10.3389/fmicb.2023.1307859

**Published:** 2023-11-30

**Authors:** Aanchal Yadav, Pallawi Kumari, Priti Devi, Jorelle Jeanne B. Adjele, Sandeep Budhiraja, Bansidhar Tarai, Rajesh Pandey

**Affiliations:** ^1^Division of Immunology and Infectious Disease Biology, INtegrative GENomics of HOst-PathogEn (INGEN-HOPE) Laboratory, CSIR-Institute of Genomics and Integrative Biology (CSIR-IGIB), Delhi, India; ^2^Academy of Scientific and Innovative Research (AcSIR), Ghaziabad, India; ^3^Indraprastha Institute of Information Technology Delhi, New Delhi, India; ^4^Centre for Food, Food Security, and Nutrition Research, Institute of Medical Research and Medicinal Plant Studies, Yaounde, Cameroon; ^5^Max Super Speciality Hospital (A Unit of Devki Devi Foundation), Max Healthcare, Delhi, India

**Keywords:** dengue infection, high and low dengue viral reads, clinical parameters, dual RNA-Seq, transcriptionally active microbes, disease severity

## Abstract

**Introduction:**

Dengue virus (DENV) is a flavivirus that has emerged as a global health threat, characterized by either asymptomatic or mild self-limiting febrile illness, but a subset of DENV outbreaks have been associated with severe disease. Studies have looked into the host immune response and dengue viral load during infection. However, it remains unknown how the active microbial isolates modulate the dengue viral infection. In this study, we demonstrate the significance of in-depth analysis of microbiota composition in the serum samples of dengue-infected patients.

**Materials and methods:**

RNA was extracted from the serum samples collected from 24 dengue positive patients. The human mapped reads generated through RNA-Sequencing (RNA-Seq) were removed, while the unmapped (non-human) reads were employed for microbial taxonomic classification using Kraken2 and Bracken2. Further, we assessed the initial blood parameters analyzing the complete blood count (CBC) profile of the patients.

**Results:**

Findings revealed differential abundance of commensals and pathogenic microbes in the early febrile period of hospitalized dengue patients, segregated into, High Viral Reads (HVR) and Low Viral Reads (LVR). The Campylobacter genus was abundant in the HVR whereas Lactobacillus dominated the LVR patients. At species level, the microbiota of HVR exhibited higher abundance of unique potential opportunistic microbes, compared to the commensal microbes’ enrichment in the LVR patients’. We hypothesize that the DENV might alter the microbiota composition as observed by the increase in preponderance of opportunistic pathogens and an absence of commensals in the HVR. The presence of commensals in the LVR might explain, i) overall lower dengue viral reads compared to the HVR, and ii) shift in lymphocytes (high) and neutrophils (low) counts; resulting in a comparatively milder clinical manifestation in this group. Our findings may help in understanding the co-infection aspect that will be important to develop dengue therapeutics and vaccines.

**Discussion:**

This study highlights the potential of the unexplored roles of the TAMs in modulating the dengue disease severity using the metatranscriptomic sequencing. This study serves to enhance our understanding of the distinctive microbial and hematologic signatures in the early infection stage that differentiate patients with high viral reads patients from those with low dengue viral reads.

## Importance of the study

Each year, India records more than a hundred thousand dengue cases resulting in immense public health burden. Here, we employed the RNA-Seq approach to explore the functional dynamics of transcriptionally active microbes (TAMs) in the serum of dengue positive individuals. We discovered that the patients who had high dengue viral load had an elevated number of opportunistic microbial species and were devoid of commensals. Contrarily, the balanced presence of pathogenic/opportunistic and commensals plausibly explains the milder clinical manifestations of LVR. In the early febrile period of Dengue, we observed a distinct demarcation of Lymphocyte and Neutrophil counts in blood parameter profiles among dengue patients between the two groups. These findings suggest that the use of blood parameters as prognostic markers, along with the identification of specific microbial patterns associated with disease severity can be used to predict disease progression.

## Introduction

Dengue virus (DENV), comprising four distinct serotypes (DENV 1–4), causes dengue infection which is one of the most important yet highly neglected tropical diseases in the world. The incidence of dengue has alarmingly increased over the past few decades, rising from 0.5 million cases in 2000 to 5.2 million in 2019 (Gutierrez-Barbosa et al., 2020). The diverse range of symptoms observed underscores the need to comprehend the underlying factor/s contributing to the severity of clinical manifestations. Notably, clinical parameters, including laboratory tests such as complete blood count (CBC), serological tests, and blood culture, along with viral antigen positivity, can be utilized to confirm the diagnosis as well as assess dengue disease severity. While an abnormal platelet count and function have been recognized as hallmarks of dengue infection, other parameters, like low lymphocyte percentage, are associated with variable disease severity (Low et al., 2011; Michels et al., 2014; Mukker and Kiran, 2018; Yadav et al., 2023).

Considering the diversity in viral genotype and clinical parameters among dengue patients, most of the studies have explored viral and host factors to define dengue infection and its impact on clinical phenotypes, seeking biomarkers of severity. Coincidentally, few studies have also reported an increased prevalence of bacterial infections, followed by dengue viral infections (JCDR, 2018; Lee J. C. et al., 2022; Yadav and Pandey, 2022). The scanty information, through case studies, gives an insight into the importance of co-infection along with the primary dengue infection (Miyata et al., 2015; de Filippis et al., 2016). COVID-19 pandemic has also highlighted the functional role of co-presence of *transcriptionally active microbes (TAMs)* as a major disease modulator (Devi et al., 2022, 2023a,b).

This pilot study focuses on the impact of microbial co-presence during dengue infection. The serum from dengue positive patients (*n* = 24) who reported to MAX Hospital, Delhi, was collected and using metatranscriptomic sequencing, microbial co-infections were comprehensively evaluated. The median age of these patients was 21.5 years with the minimum and maximum age of 3 and 68 years, respectively. Also, 66.7% (*n* = 16) of the patients were male as compared to 33.3% (*n* = 8) females.

## Materials and methods

### Patient sample collection

The study was carried out at CSIR-Institute of Genomics and Integrative Biology (CSIR-IGIB) in collaboration with MAX Healthcare Hospital, Delhi, India. Dengue positive patients (*n* = 24) who reported to MAX Healthcare Hospital, Delhi, India, were recruited into this study. After collecting blood from these patients, serum was separated from the blood and tested for dengue NS1Ag. All samples had a ratio of >1.00 for the Dengue NS1Ag Test. Dengue viral detection was done from the serum sample collected by the paramedical staff at the hospital on the day of reporting. Bio-Rad Platelia Dengue NS1 Ag test was used for detecting DENV using the Dengue NS1 Antigen Test (ELISA).

### Serum RNA isolation, library preparation and sequencing

Viral RNA was isolated from the serum using QIAamp RNA Blood Mini Kit (cat. No. 52304) which was used for RNA-Seq. The library preparation protocol for RNA-Seq has been previously published from our lab (Yadav et al., 2023). Briefly, a total of 250 ng of RNA were utilized for library preparation using the Illumina TruSeq Stranded Total RNA Library Prep Gold (Illumina, Cat. No. 20020598). The final library was quantified using a Qubit double-stranded DNA (dsDNA) high-sensitivity (HS) assay kit (Thermo Fisher Scientific; catalog no. Q32854). The quality of cDNA libraries was checked using the Agilent 2100 Bioanalyzer. A final loading concentration of 650 pM was used for sequencing using NextSeq 2000, with paired-end 2 × 151 reads.

### RNA pre-processing and bioinformatic analysis

Base call files from sequencer were converted into FASTQ format using bcltofastq. FastQC and Trimmomatic-0.36 were used to check the quality of the reads and remove adapters as well as low quality reads (Bolger et al., 2014), respectively. The high quality trimmed reads were aligned to the reference human transcript using HISAT2 to remove human host RNA reads. For the human unaligned (microbial) reads, Kraken2 (Wood et al., 2019) and Bracken2 (Lu et al., 2017) were used to infer microbial communities. Bracken2 (Bayesian Reestimation of Abundance with KrakEN) was performed to identify the Dengue reads across the samples, using the non-human reads from the RNA-seq data. The Kraken2 database was used to create a Bracken-compatible database using the brackenbuild function, and the Kraken2 report files for each sample were run against the Bracken database using the bracken function for the phylum, genus and the species level information. Taxonomic Diversity Analysis through Alpha and Beta diversity were performed using the phyloseq (v1.27.2) and vegan (v2.5–4) packages in R (v3.4.3). To further analyze the beta diversity, we utilized Bray-Curtis distance matrices and performed principal coordinate analysis (PCoA), where PC1 accounted for 24.31% of the variance and PC2 for 10.22%.

### Statistical analysis

Statistical analysis for clinical parameters were done through Mann–Whitney test using Graph pad prism. For Beta diversity analysis, PERMANOVA/adonis2 test in R (using the Vegan (v2.5–4) package) was calculated to determine the statistical significance. To remove count data bias, we employed MetagenomeSeq’s Cumulative Sum Scale (CSS) algorithm for data normalization. STAMP software with Welch’s test, effect sizes, and 95% confidence intervals was used to screen statistical significance for common species across HVR and LVR patients.

### Dengue serotype classification

Trimmed Fastqs were mapped to DENV Serotypes 1–4, using HISAT2. The final serotype of a particular sample is determined by the serotypes with the highest percentages in that sample. BAM was converted into consensus FASTA using bcftools.

### Availability of dataset

The datasets generated during the current study are available in the NCBI SRA under the access number PRJNA955953.

## Results

### Change in hematological characteristics (lymphocytes and neutrophils) as predictive markers for dengue disease outcome in early febrile period

RNA-Seq generated 12,964,528 raw sequencing reads, which were categorized into two main categories: Human and Non-human reads. The non-human reads were analyzed using Kraken2 followed by the Bracken2, a k-mer based taxonomic classification tool, for identification of microbial presence using Bayesian re-estimation of abundance. In total, 24 samples generated an average of ~2,775,261 microbial reads/sample. Our analysis revealed an intriguing unequal distribution of dengue virus reads, which prompted us to divide them into two distinct groups: High viral reads (HVR) and Low viral reads (LVR) with 12 samples each (Supplementary file S1). For HVR and LVR patients, the median ages were 25 and 36, respectively.

**Figure 1:**
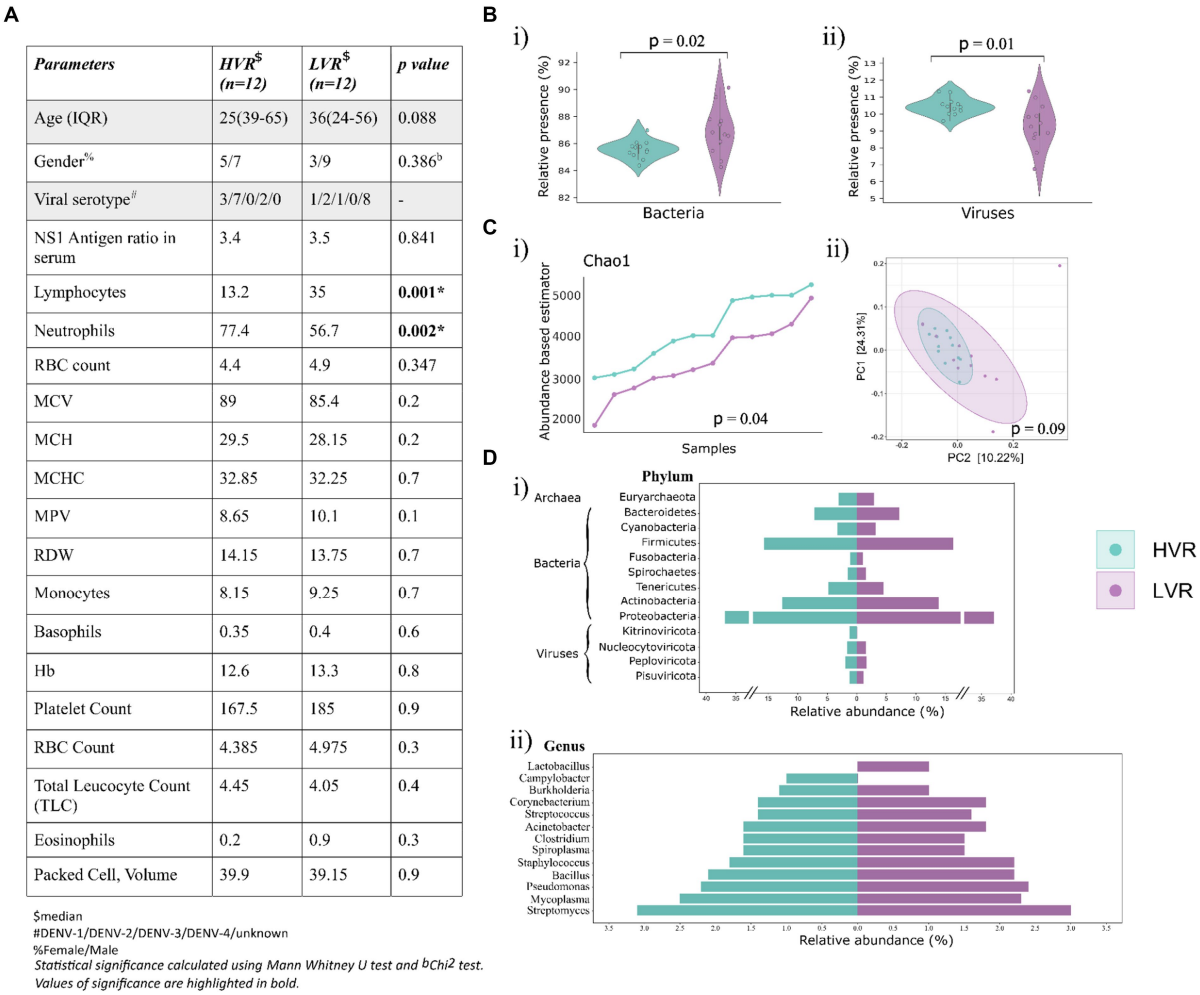
Dengue patients’ clinical data overview, microbial abundance and diversity. **(A)** Key demographic features, viral serotype and clinical characteristics (CBC) of the dengue patients. **(B)** Violin plots showing the relative abundance of **(i)** bacteria, and **(ii)** viruses. **(C)** Visualization of alpha and beta diversity, **(i)** Chao-1 (Abundance-based estimator) alpha diversity index with *p*-values calculated by the Kruskal Wallis test, **(ii)** Beta diversity wherein Principal Coordinate Analysis (PCoA) shows the differential composition of the microbes across HVR and LVR. **(D)** Illustration of percent relative abundance at **(i)** phyla and **(ii)** genera level identified in the two groups.

Further, we identified DENV Serotypes from the non-human RNA-seq reads in 16/24 samples, of which 12 samples with high DENV reads were serotype positive, while 4 samples from the LVR were positive for serotype identification. Simultaneously, we conducted a comprehensive analysis of CBC parameters in patients corresponding to the two groups (Figure 1A). Analysis allowed us to explore how these parameters were influenced by dengue infection. In terms of platelet and total leukocyte count, which are considered critical hematological features for predicting dengue disease outcomes, no significant differences have been found. Can lymphocytes and neutrophils be used as potential predictive markers for disease progression, since we observed a significant difference for these two parameters? In contrast to the LVR group, lymphocytes and neutrophils in the HVR patients displayed low and high values, respectively (Figure 1A). The shift in lymphocytes count has previously been associated with dengue infection, studies have highlighted that the patients with early disease had significantly lower lymphocyte counts (Sigera et al., 2019). The evidence, however, is still up for debate as to how neutrophils contribute to the disease progression. While a few studies reported early-phase neutropenia in dengue patients, a higher neutrophil percentage predominantly in the first 5 days of the fever has been revealed by a retrospective study (Chaloemwong et al., 2018). The study additionally identified a negative correlation between decreased neutrophil and increased lymphocytes count, emphasizing the neutrophil to lymphocyte ratio as > 1 on the first 5 days. Further research is required to understand the functional implications of neutrophil levels and dengue disease outcomes.

### Overview of TAMs diversity and phyla and genera level microbial community composition

To gain insights into the microbial genomes alongside the dengue genome, we focused on analyzing the non-human reads. Using taxonomic classification analysis, we mapped 4,859 TAMs from the bracken database.

The analysis revealed the presence of bacterial, viral, and archaea bacterial genomes. Bacteria was the predominant microbial community (77.1%), followed by viruses (18.7%) and archaea (4.25%) (Figure 1B). A significant distribution in the viral and bacterial populations was observed across the two groups, but not for archaea (Supplementary Figure S1A). We further proceeded to compare the alpha and beta diversity metrics of patient microbiomes in the HVR and LVR. Incidentally, we did not observe significant differences in within-sample (alpha) and between-sample (beta) diversities of the microbiomes between HVR and LVR patients. However, there was a notable difference in the Chao1 index, statistically significant (*p* = 0.04, Kruskal-Wallis test) (Figure 1C) (Supplementary Figure S1B). Beta diversity analysis also showed non-significant clustering patterns between the two groups when visualized in a PCoA plot.

For in-depth analysis of the microbial composition, we focused on understanding the relative abundance of phyla and genera in both the groups, which showed striking similarities (Supplementary Table S2). Notably, we observed that *Proteobacteria* (36.8% in HVR vs. 37.1% in LVR), *Actinobacteria* (12.6% in HVR vs. 13.8% in LVR), and *Firmicutes* (15.7% in HVR vs. 16.3% in LVR) were prevalent and equally distributed phyla in both HVR and LVR. In terms of viral phyla, *Nucleocytoviricota*, *Peploviricota*, and *Pisuviricota* exhibited no significant differences between the HVR and LVR, except for the phylum *Kitrinoviricota*, which dengue virus belongs to (Figure 1Di).

Further narrowing down the analysis for genera, *Lactobacillus* showed abundance in the LVR (1% LVR vs. <1% HVR), while *Campylobacter* exhibited abundance in the HVR (1% HVR vs. <1% LVR). The *Campylobacter* genus are a diverse group of bacteria, mostly regarded as important human pathogens (Facciolà et al., 2017). Contrariwise, the *Lactobacillus* comprises the beneficial species that are generally a major part of human microbiota, including the digestive and female genital system (Fijan, 2014). However, we also observed shared characteristics in the abundance of certain genera among both dengue sub-groups (Figure 1Dii). The genera characterized by higher relative abundance differences in both the groups are highlighted in Table 1. Although not so stark, we uncovered significant differences in the prevalence of commensal and opportunistic microbial taxa at the genera-level between HVR and LVR groups.

**Table 1:** Distribution of genus present at >1% relative abundance in two groups.

Genus	HVR (%)	LVR (%)
*Streptomyces*	3.1	3
*Mycoplasma*	2.5	2.3
*Pseudomonas*	2.2	2.4
*Bacillus*	2.1	2.2
*Staphylococcus*	1.8	2.2
*Clostridium*	1.6	1.5
*Acinetobacter*	1.6	1.8
*Spiroplasma*	1.6	1.5
*Streptococcus*	1.4	1.6
*Corynebacterium*	1.4	1.8
*Burkholderia*	1.1	1

### Differential presence of commensals and opportunistic active microbial species reveals their putative role in disease trajectory

The presence of functionally distinct genera in the two groups intrigued us to delve deeper at the species level. To ensure stringency of our findings, the species with a relative cumulative abundance of <0.05% and presence in <50% of the samples were excluded, resulting in a dataset of 607 species.

#### From common TAMs…

**Figure 2:**
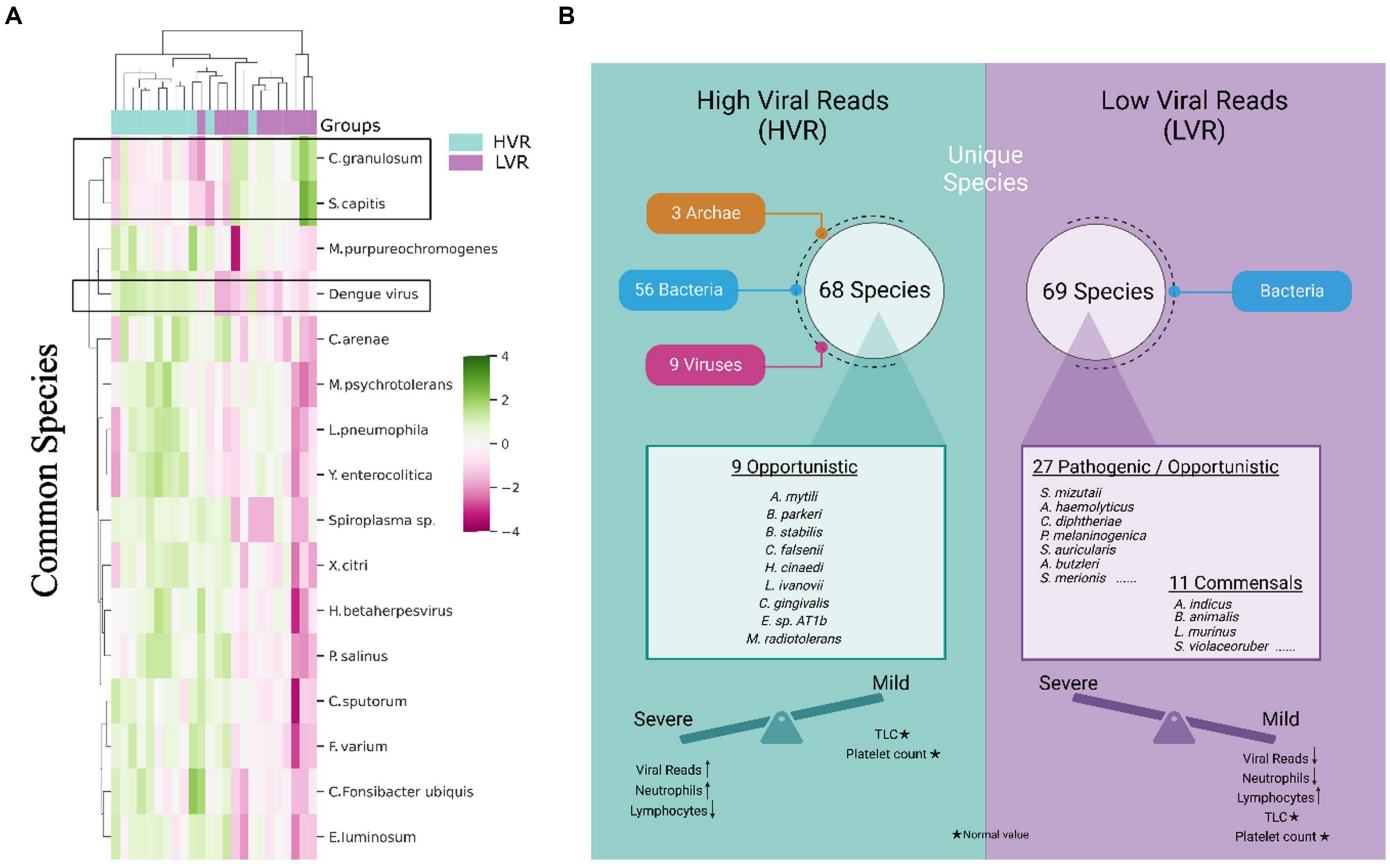
Transcriptionally active microbial species spectrum across the HVR and LVR Dengue patients. **(A)** Heatmap represents the differential abundance of 16 significant common bacterial species across the 2 groups. **(B)** Functional illustration highlighting the unique microbial species present in the HVR and LVR and their role toward shifting the disease trajectory to mild or severe along with the clinical information.

We specifically examined the species that were shared between the HVR and LVR. Of the total species identified, we found 470 common species between the groups. Using the STAMP tool for significance, 16 species showed notable differences. Heatmap depicts the varying abundance of these species in the HVR and LVR (Figure 2A). Interestingly, two species, *Cutibacterium granulosum* and *Staphylococcus capitis*, were found to be abundant in the LVR, while the remaining 14 species, including *Dengue virus*, showed abundance in the HVR. It is worth mentioning that certain species identified in our study, such as *Fusobacterium varium* and *Staphylococcus capitis*, have been reported as pathogenic or opportunistic bacteria associated with human infections (Mayslich et al., 2021; Chong et al., 2022; Lee S. J. et al., 2022). Although *Campylobacter sputorum* itself is not directly linked to disease, the *Campylobacter* genus has been associated with clinical conditions such as watery stools with fever, abdominal pain, vomiting, and dehydration, all of which are symptoms commonly observed in dengue infection (Mazaheri et al., 2016).

#### …to unique TAMs

The identification of potential TAMs as biomarkers that could explain the association between HVR and an increased disease progression needs to be evaluated. We investigated the unique microbial species between two groups. In the HVR, we identified 68 unique species, while the LVR had 69 unique species. Interestingly, we observed that all 69 species in the LVR belonged to bacteria, whereas in the HVR, there were 56 bacteria, along with 9 viruses and 3 archaea. Findings revealed that the HVR was predominantly characterized by the presence of opportunistic or potential pathogenic bacteria, namely *Capnocytophaga gingivalis*, *Burkholderia stabilis*, *Bacillus cytotoxicus*, and few emerging pathogens that are commonly reported in immunocompromised patients, including *Methylobacterium radiotolerans*, *Listeria ivanovii*, *Borrelia parkeri*, *Helicobacter cinaedi*, *Corynebacterium falsenii*, and *Exiguobacterium* sp. *AT1b* (Ehrmann et al., 2016; Cordovana et al., 2019; Cairo et al., 2022). Surprisingly, the presence of commensals, such as *Bifidobacterium animalis*, *Lactobacillus crispatus*, *Lactobacillus murinus*, *Neisseria elongata*, *Neisseria subflava*, and *Streptomyces violaceoruber* was found within the LVR whereas such commensal species were completely absent in the HVR (Jungersen et al., 2014; Kim et al., 2019; Pusparajah et al., 2021) (Figure 2B). Notably, species such as *Bifidiobacter animalis* and *Lactobacillus murinus* in the LVR function as probiotics, known to inhibit pathogens (Jungersen et al., 2014; Huang et al., 2016).

The LVR exhibits a noteworthy balance between opportunistic and commensal TAMs, which, coupled with decreased dengue viral reads, potentially plays a role in mitigating the severity of the disease. Additionally, we noticed an increased presence of various rare species, including marine and plant species, in the HVR, which might suggest a correlation with patients’ dietary habits. Overall, our functional characterization of transcriptionally active opportunistic/pathogenic and commensal microbial species helps us understand the underlying reasons for the clinical differences between HVR and LVR patients in terms of disease severity.

## Discussion

With the lessons learnt from the COVID-19 pandemic, it is extremely crucial to understand the role of co-presence/co-infection of microbes in addition to primary infection agent, with disease severity. In the present pilot study, we investigated 24 dengue patients with high and low dengue viral load. To the best of our knowledge, this is the first study which reveals the dynamics of TAMs in the serum RNA of dengue patients, captured through host RNA-seq. Our modified meta-transcriptomic strategy identified higher abundance of rare and opportunistic bacterial species in HVR.

Of note, studies in other viral infectious diseases, influenza and COVID-19, have revealed functional impact of microbes in modulating disease trajectory. Few studies have explored the microbial community structure in *Aedes* mosquito, a vector for dengue virus (Rodpai et al., 2023). Till now, there is no published metatranscriptome study on dengue co-infection within the human host, except for a few studies highlighting concurrent bacterial co-infection in adult patients with dengue fever (Lee et al., 2005; Trunfio et al., 2017).

Our results revealed a remarkable difference in the unique microbial community between HVR and LVR. The HVR harbors a higher abundance of rare species. The presence of transcriptionally active isolates of opportunistic microbial species might be playing a modulatory role in disease severity, as exemplified by the presence of *Burkholderia stabilis*, *Capnocytophaga gingivalis*, and *Arcobacter mytili* in the HVR, where *B. stabilis* is an opportunistic pathogen that cause nosocomial bloodstream infections (Otağ et al., 2005). Also, *C. gingivalis* has been described as a multidrug-resistant bacteria, associated with acute exacerbation of chronic obstructive pulmonary disease (COPD) in an immunocompetent patient (Ehrmann et al., 2016). The LVR, on the other hand, exhibited a balanced presence of both pathogenic and commensal species, of which commensals were completely absent in HVR. Along with other commensals from intestinal flora (*Streptomyces violaceoruber, Bifidobacterium animalis*), vagina (*Gardnerella vaginalis*), skin (*Auricoccus indicus*), and oral cavity (*Propionibacterium* sp. *oral taxon 193*), *Lactobacillus*, which are probiotics, at both the genus and species level, were the unique species of LVR. This may aid the host to fight off the virus thereby ameliorating severe disease conditions.

## Conclusion

In this study, the serum samples were predominantly sourced from 24 dengue-positive patients from a single hospital within a specific region to investigate microbial patterns during dengue infection. It’s important to acknowledge that this approach has certain limitations. While this choice reduces variability within the samples, it also limits the broader applicability of our findings. These findings will be strengthened with future studies to examine the functional role of co-presence of TAMs in a larger and diverse dengue patient cohort, as well as in the blood isolated RNA, where the DENV replication occurs. Additionally, a longitudinal sampling approach of dengue patients would have provided valuable insights into the dynamic changes in active microbial populations throughout the disease trajectory. Longitudinal sampling of the dengue patients may further enhance the scope to understand the dynamics of active microbial populations during the disease trajectory. Despite that, the present study, which might point to the transition of disease trajectory (mild or severe) by the co-presence of various microbial communities along with the primary dengue virus and their distribution across the two groups, provided crucial insight into this unexplored yet important role of TAMs in dengue viral infection.

## Data availability statement

The datasets presented in this study can be found in online repositories. The names of the repository/repositories and accession number(s) can be found in the article/Supplementary material.

## Ethics statement

The studies involving humans were approved by the Institutional Ethics Committee of both CSIR-Institute of Genomics and Integrative Biology, and Max Super Specialty Hospital, under the approval number CSIR-IGIB/IHEC/2020-21/01. The studies were conducted in accordance with the local legislation and institutional requirements. Written informed consent for participation in this study was provided by the participants’ legal guardians/next of kin.

## Author contributions

RP: Conceptualization, Funding acquisition, Resources, Supervision, Visualization, Writing – review & editing. AY: Data curation, Formal analysis, Investigation, Visualization, Writing – original draft. PK: Formal analysis, Methodology, Visualization, Writing – original draft. PD: Data curation, Investigation, Supervision, Writing – original draft. JA: Resources, Writing – review & editing. SB: Resources, Writing – review & editing. BT: Resources, Writing – review & editing.

## Supplementary material

SUPPLEMENTARY FILE S1 Kraken output file showing a diverse group of bacteria and viruses for all the 24 samples, with 12 samples having high dengue virus reads and the rest showing lower dengue viral reads based on RNA-seq.

SUPPLEMENTARY FIGURE S1 **(a)** Relative abundance of archaeal reads in our cohort **(b)** Alpha diversity indices showing bacterial species richness (Shannon) and evenness (Simpson) across the patients in HVR and LVR.

SUPPLEMENTARY TABLE S2 Taxonomic communities at the level of phylum and genus, identified through Bracken in HVR and LVR cohort.
