# Supplementary material for: Dual RNA-Seq reveals transcriptionally active microbes (TAMs) dynamics in the serum of dengue patients associated with disease severity
Source: Front Microbiol. 2023 Nov 30;14:1307859. doi: 10.3389/fmicb.2023.1307859 (PMC10723774; doi:10.3389/fmicb.2023.1307859)

**Supplementary File S1:** Kraken output file showing a diverse group of bacteria and viruses for all 24 samples, with 12 samples having high dengue virus reads.


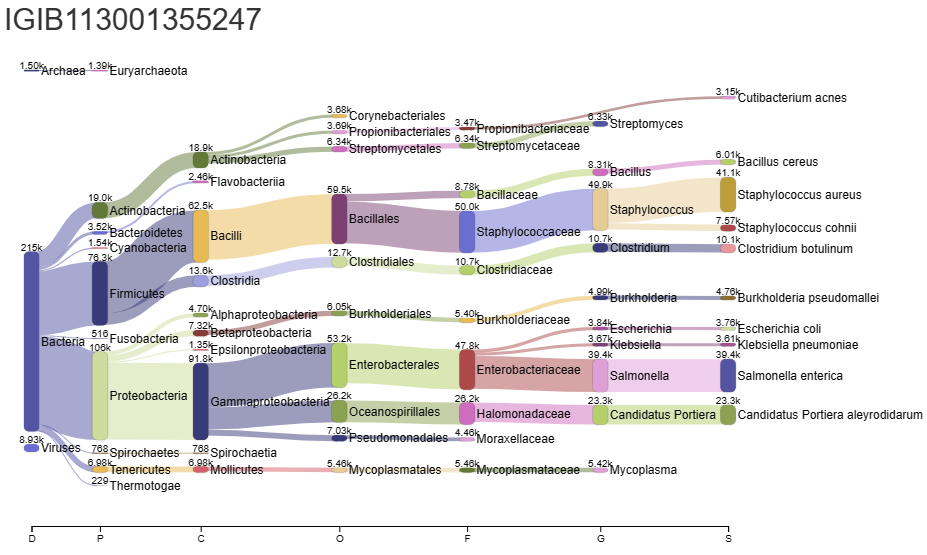


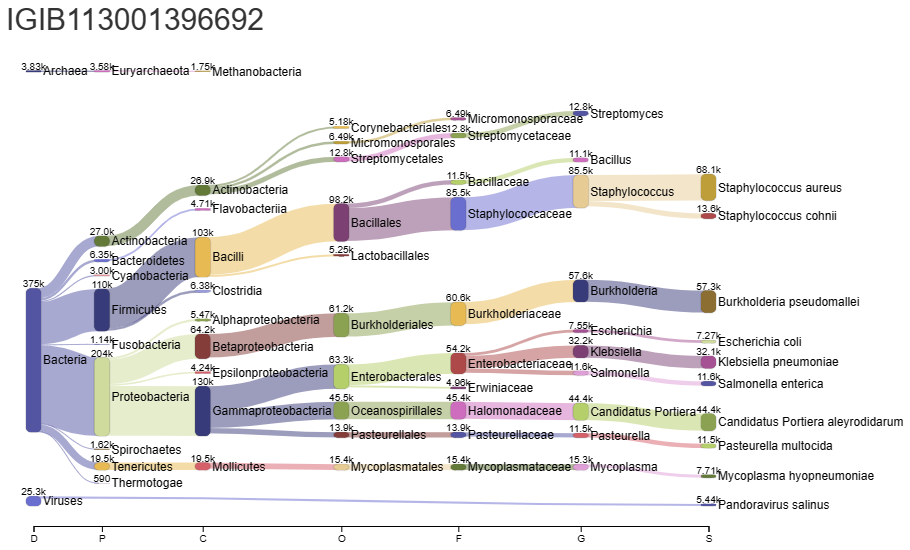


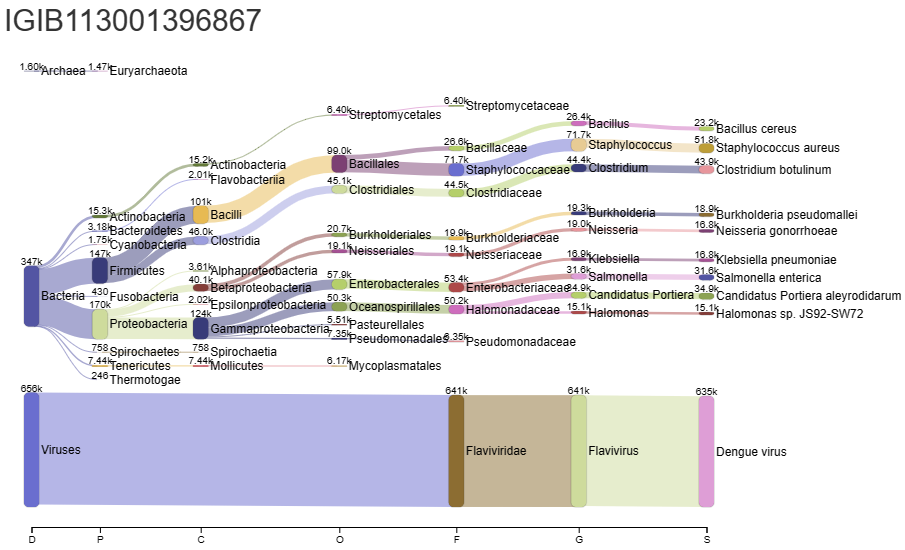


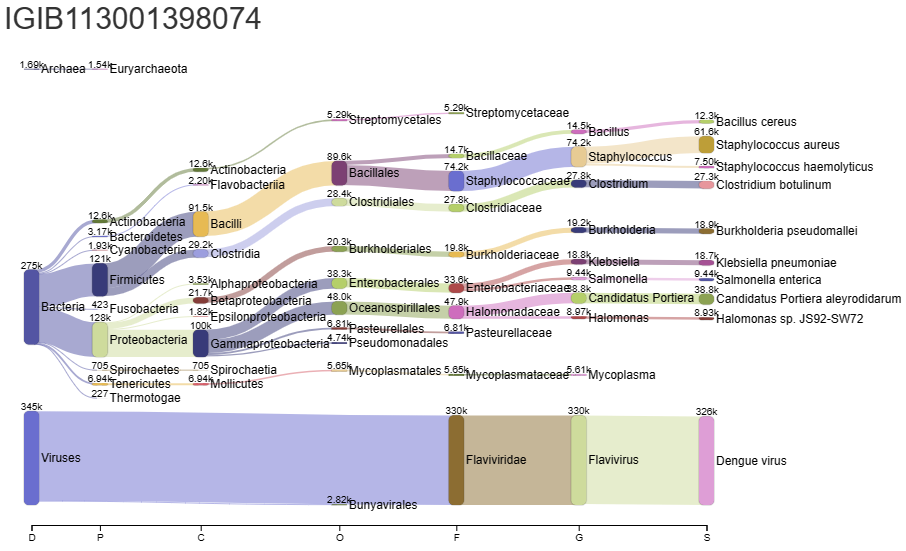


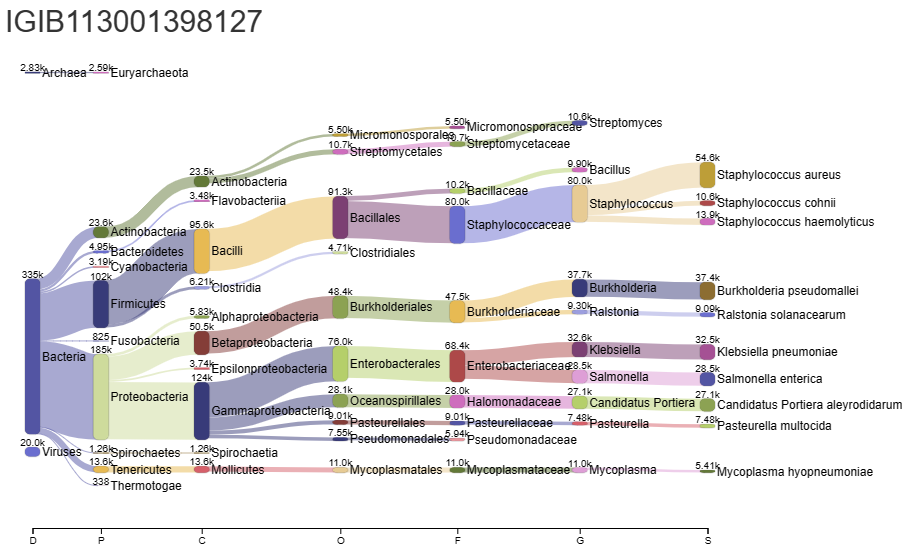


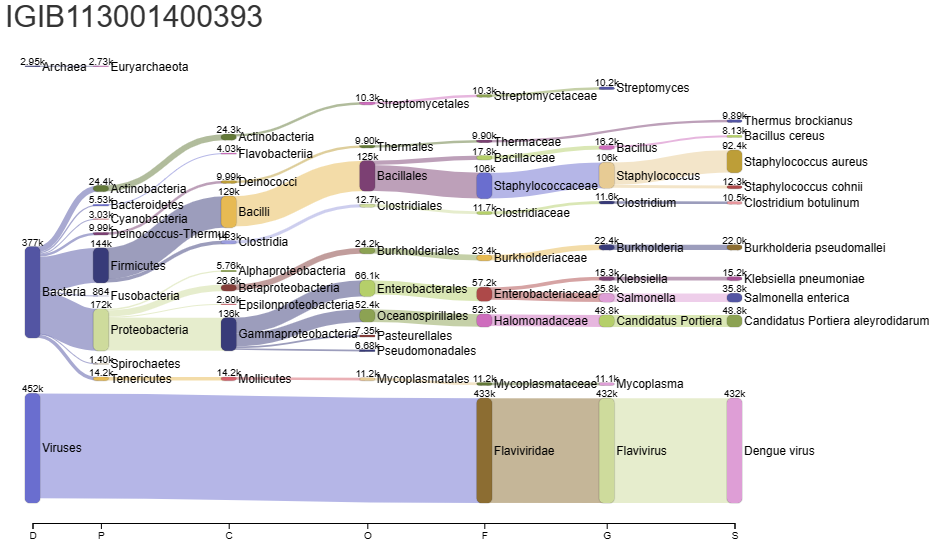


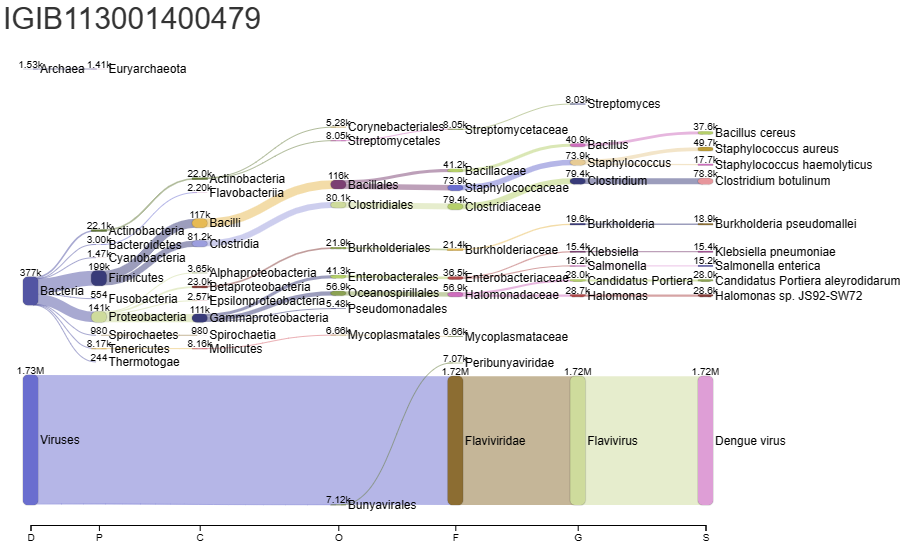


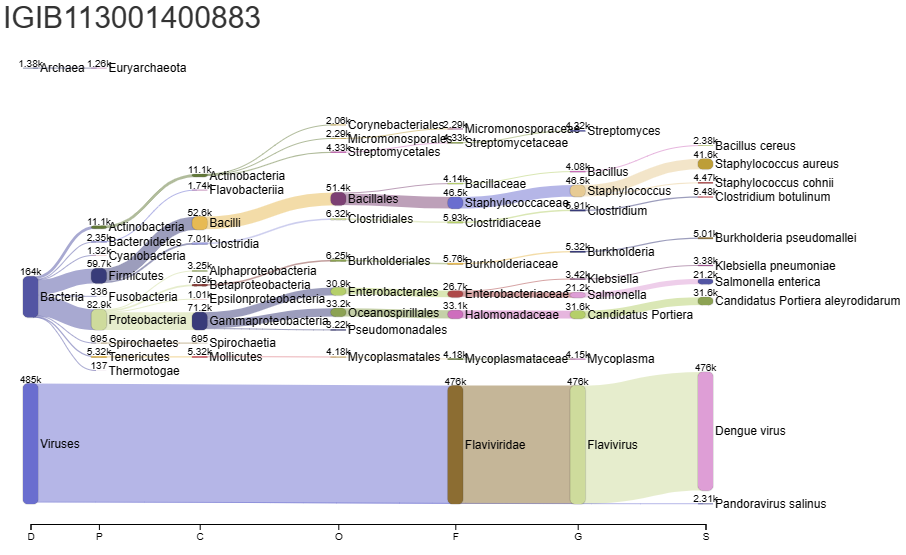


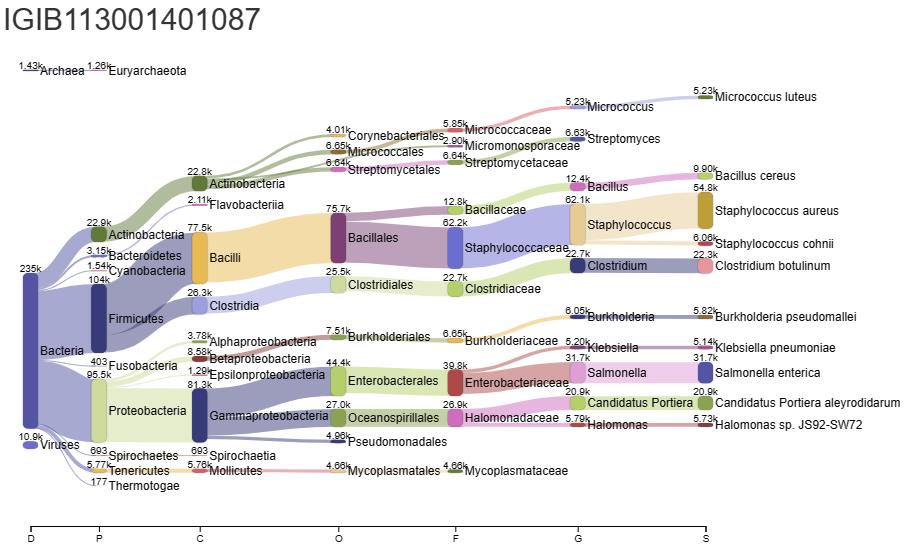


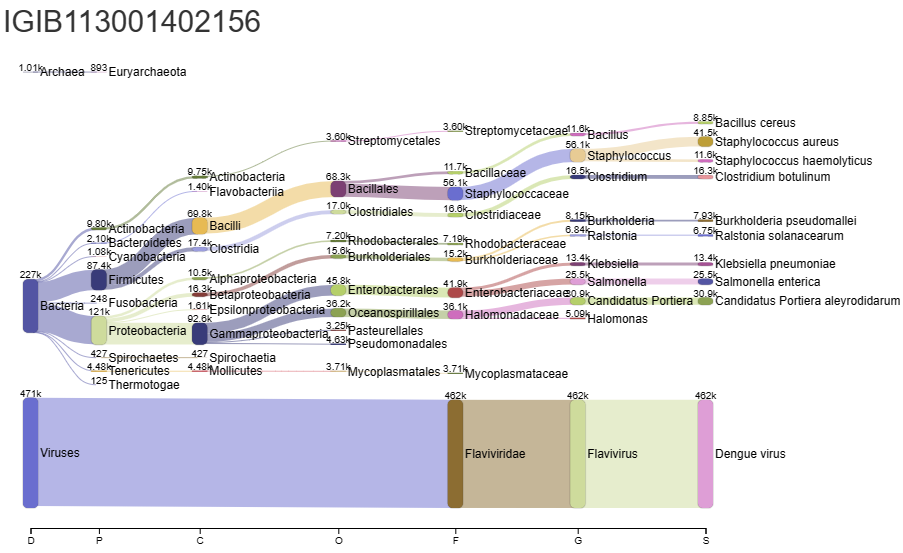


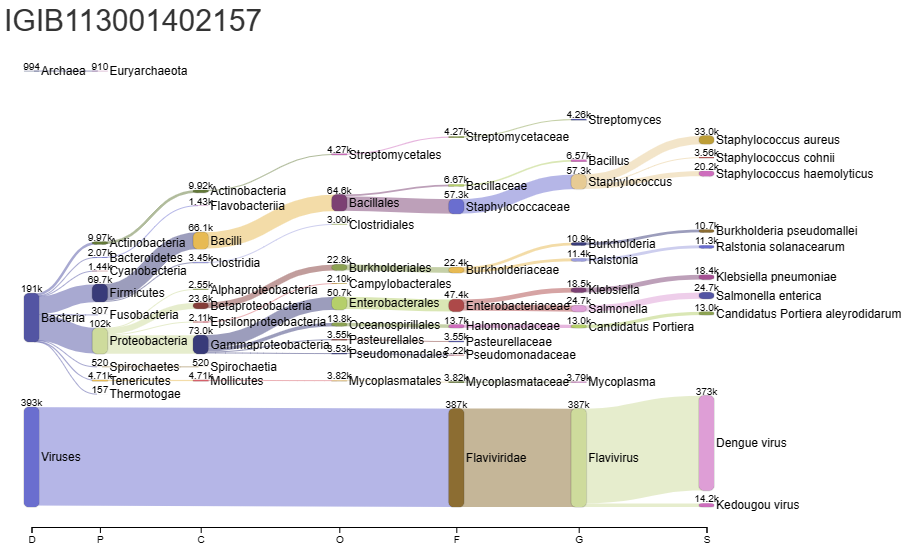


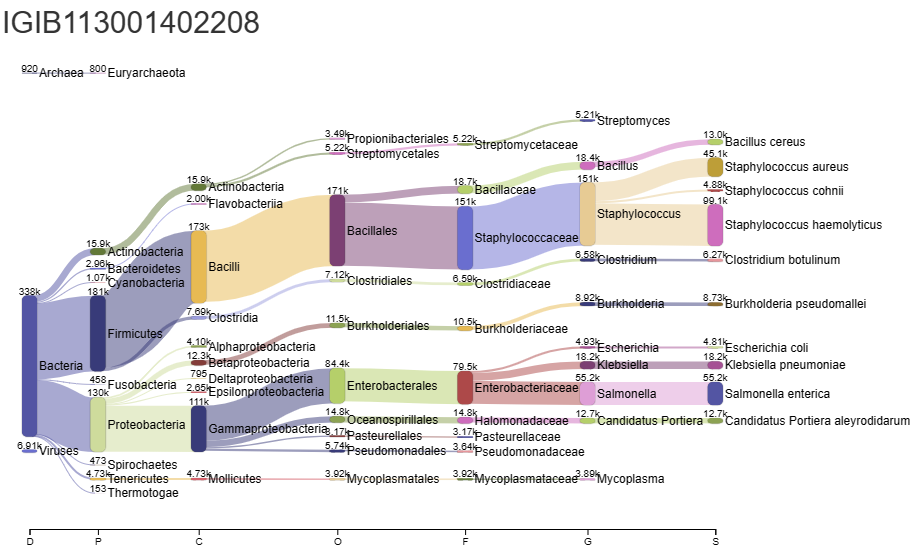


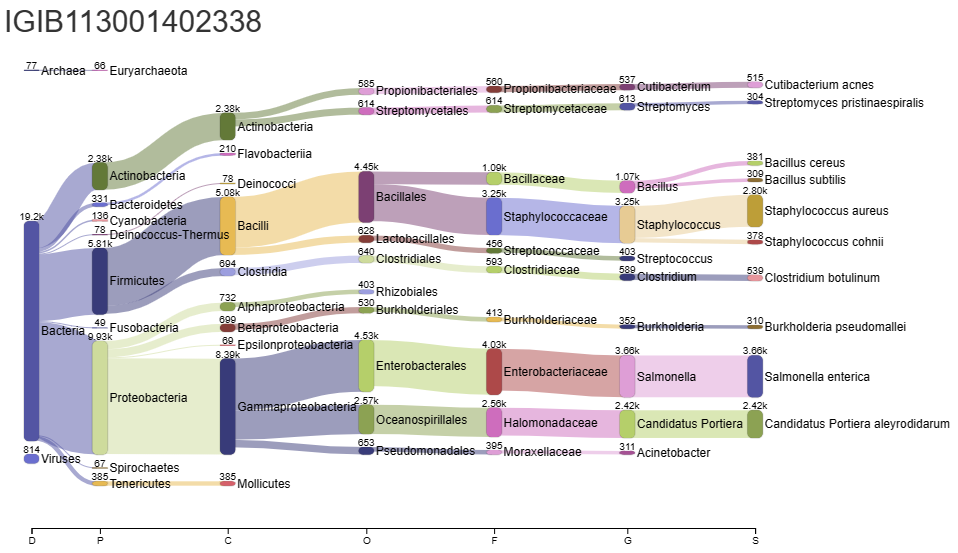


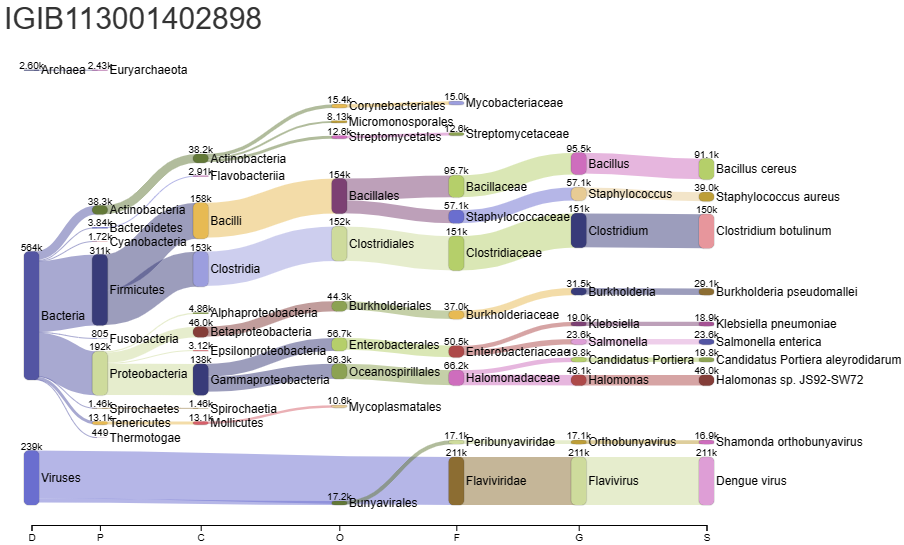


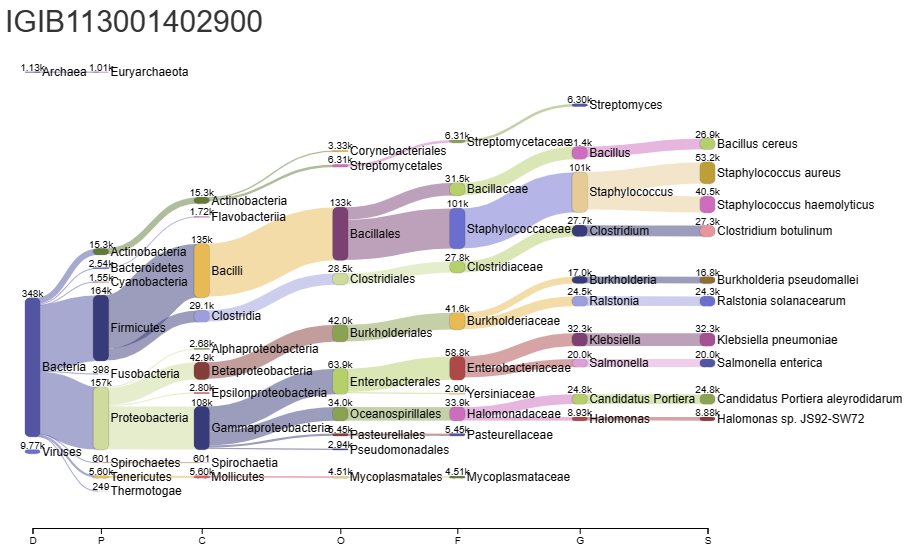


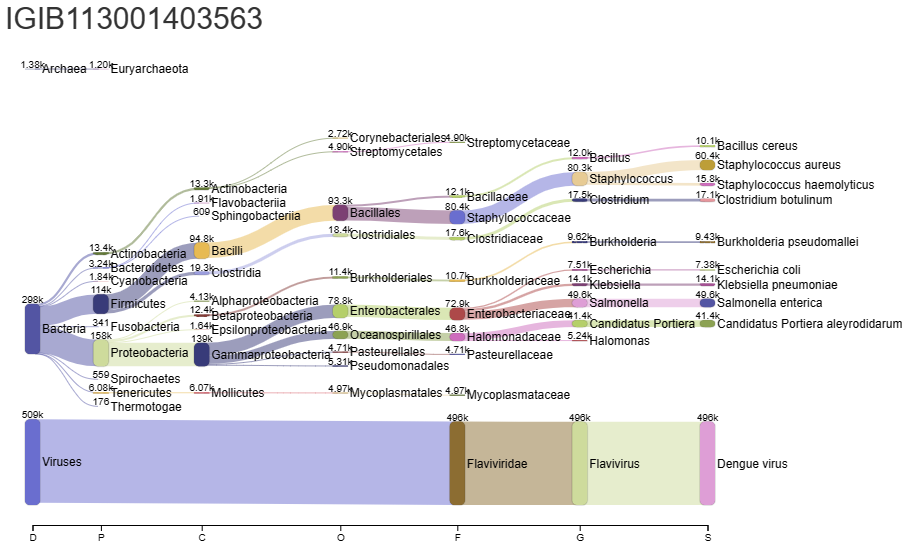


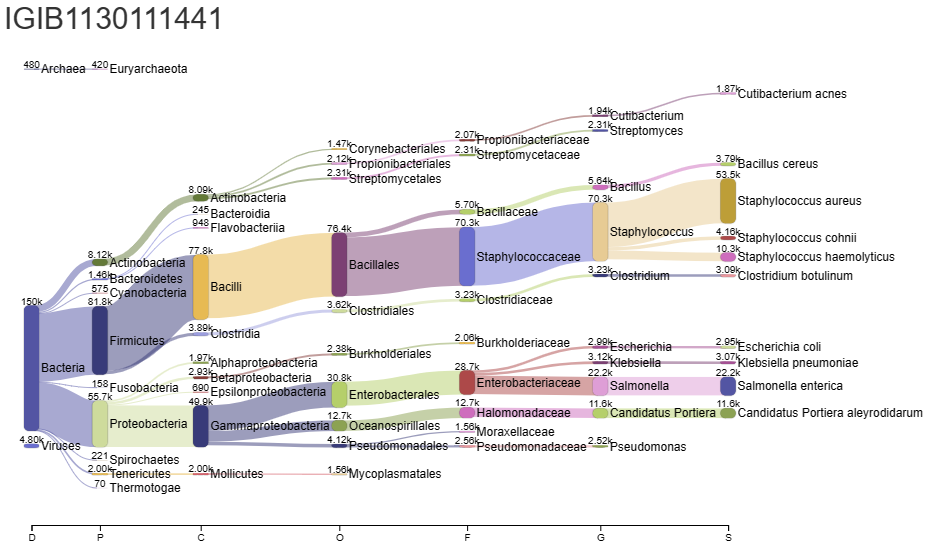


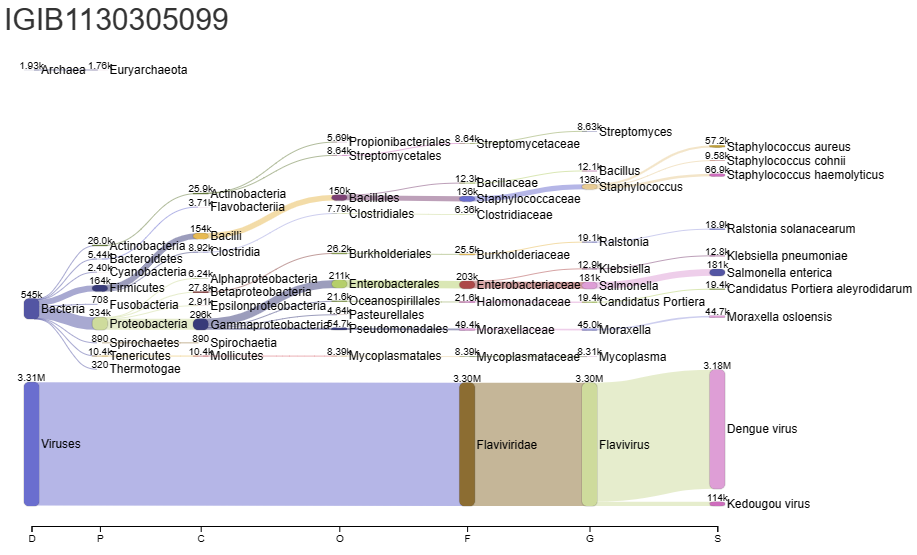


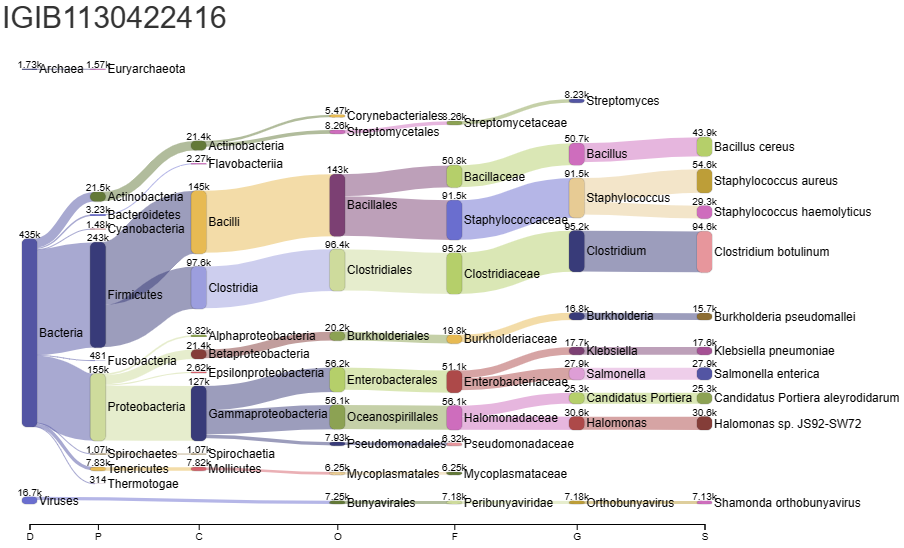


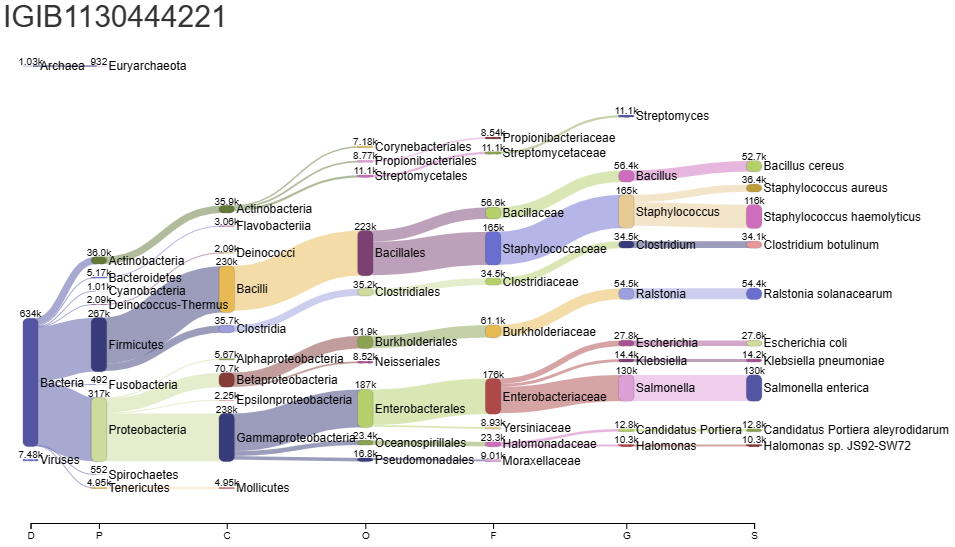


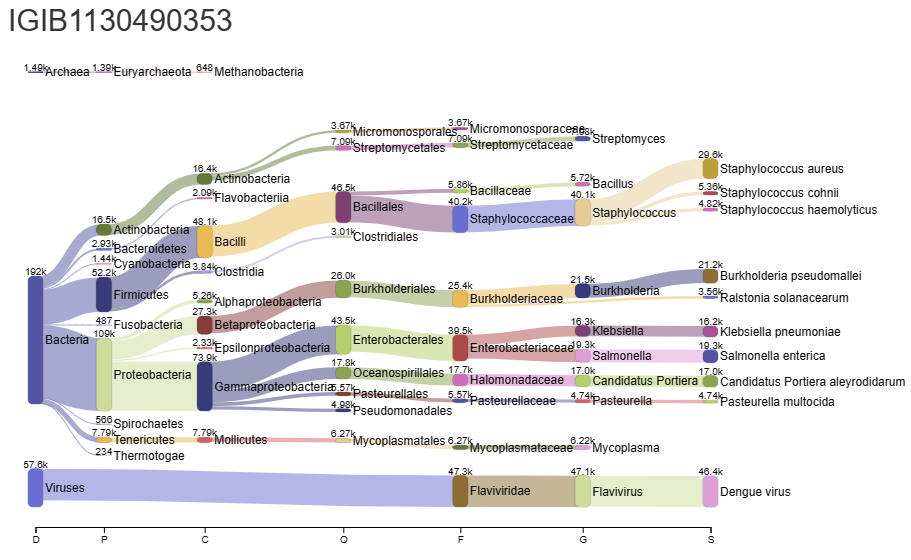


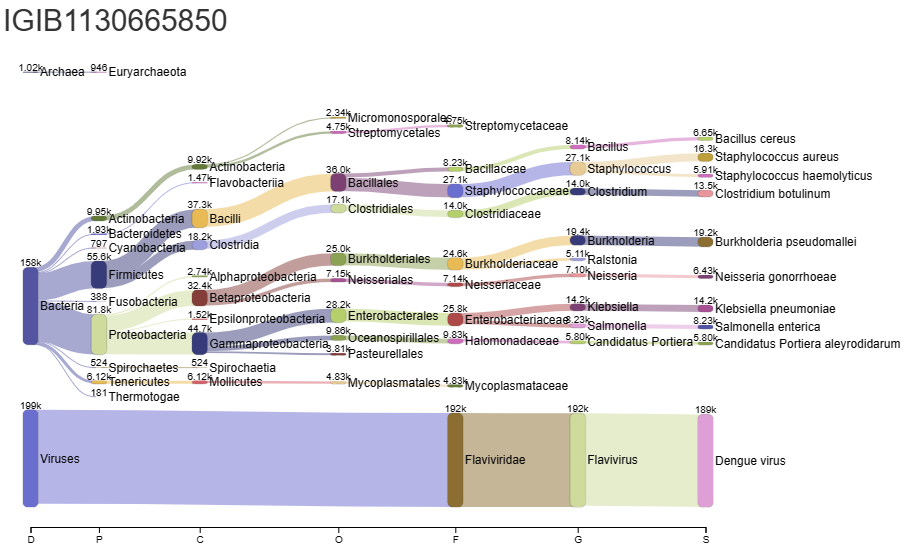


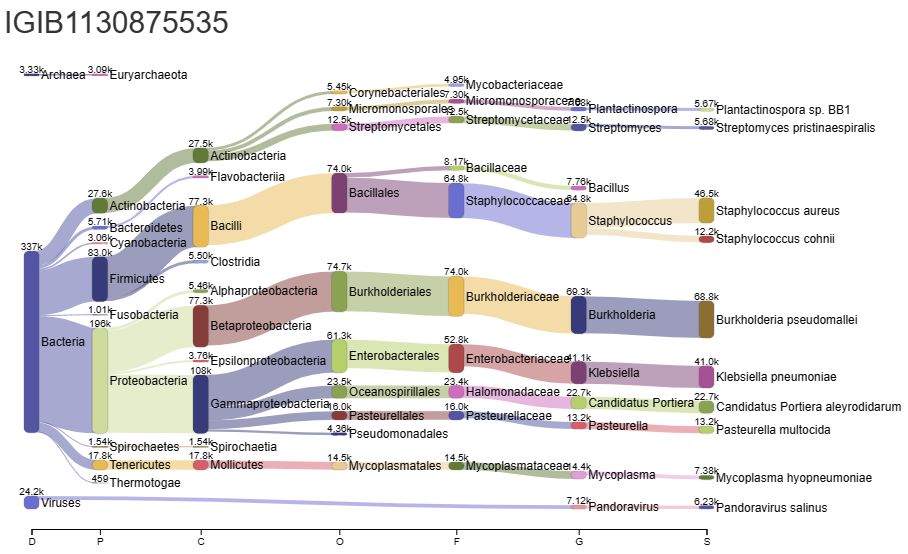


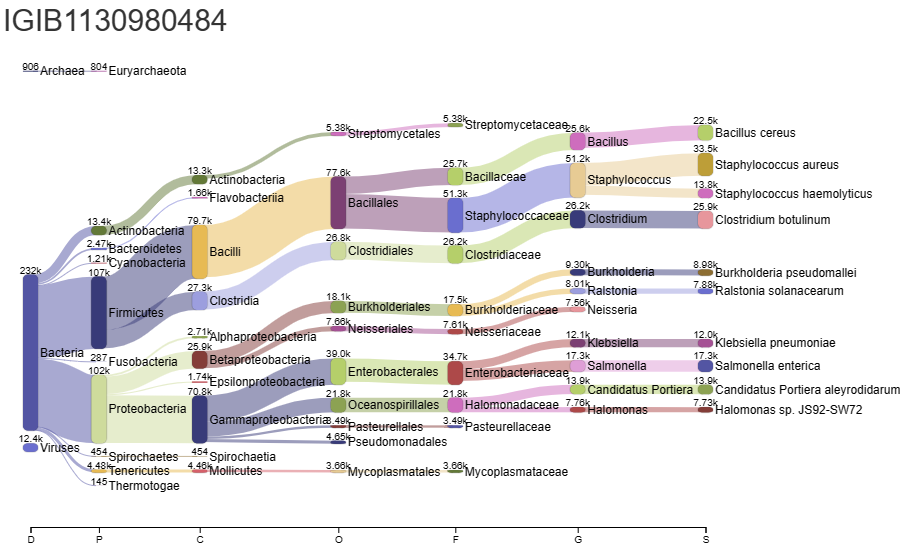

Supplement: SUPPLEMENTARY FILE S1 — Kraken output file showing a diverse group of bacteria and viruses for all the 24 samples, with 12 samples having high dengue virus reads and the rest showing lower dengue viral reads based on RNA-seq. [file Data_Sheet_1.docx]
