# Supplementary material for: Dual RNA-Seq reveals transcriptionally active microbes (TAMs) dynamics in the serum of dengue patients associated with disease severity
Source: Front Microbiol. 2023 Nov 30;14:1307859. doi: 10.3389/fmicb.2023.1307859 (PMC10723774; doi:10.3389/fmicb.2023.1307859)
Supplement: SUPPLEMENTARY FIGURE S1 — (a) Relative abundance of archaeal reads in our cohort (b) Alpha diversity indices showing bacterial species richness (Shannon) and evenness (Simpson) across the patients in HVR and LVR. [file Image_1.pdf]

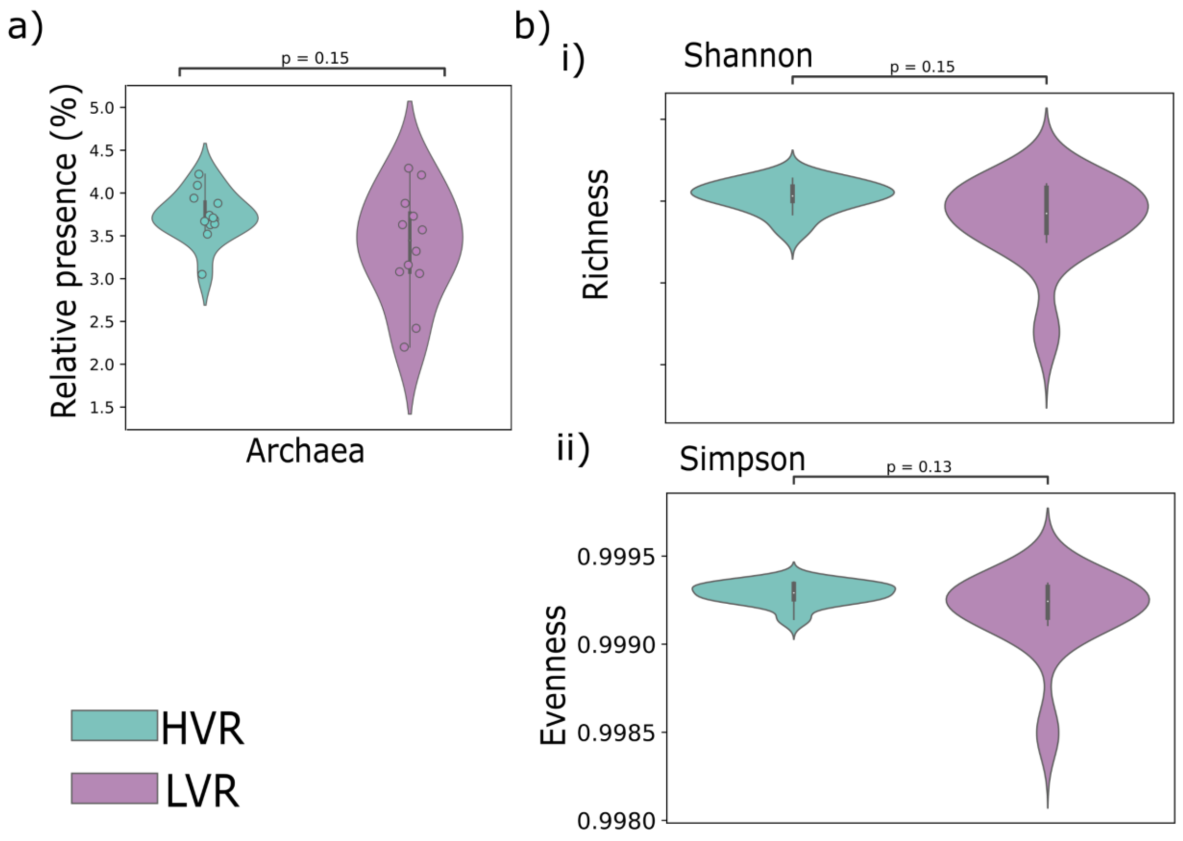

Supplementary Figure S1: a) Relative abundance of archaeal reads in our cohort **b)** Alpha diversity indices showing bacterial species richness (Shannon) and evenness (Simpson) across the patients in HVR and LVR.
